# Classification Methods Based on Complexity and Synchronization of Electroencephalography Signals in Alzheimer’s Disease

**DOI:** 10.3389/fpsyt.2020.00255

**Published:** 2020-04-07

**Authors:** Sou Nobukawa, Teruya Yamanishi, Shinya Kasakawa, Haruhiko Nishimura, Mitsuru Kikuchi, Tetsuya Takahashi

**Affiliations:** ^1^ Department of Computer Science, Chiba Institute of Technology, Narashino, Japan; ^2^ AI & IoT Center, Department of Management Information Science, Fukui University of Technology, Fukui, Japan; ^3^ Graduate School of Applied Informatics, University of Hyogo, Kobe, Japan; ^4^ Research Center for Child Mental Development, Kanazawa University, Kanazawa, Japan; ^5^ Department of Psychiatry & Behavioral Science, Kanazawa University, Ishikawa, Japan; ^6^ Department of Neuropsychiatry, University of Fukui, Yoshida, Japan

**Keywords:** Alzheimer’s disease, electroencephalography, complexity, functional connectivity, machine learning

## Abstract

Electroencephalography (EEG) has long been studied as a potential diagnostic method for Alzheimer's disease (AD). The pathological progression of AD leads to cortical disconnection. These disconnections may manifest as functional connectivity alterations, measured by the degree of synchronization between different brain regions, and alterations in complex behaviors produced by the interaction among wide-spread brain regions. Recently, machine learning methods, such as clustering algorithms and classification methods, have been adopted to detect disease-related changes in functional connectivity and classify the features of these changes. Although complexity of EEG signals can also reflect AD-related changes, few machine learning studies have focused on the changes in complexity. Therefore, in this study, we compared the ability of EEG signals to detect characteristics of AD using different machine learning approaches one focused on functional connectivity and the other focused on signal complexity. We examined functional connectivity, estimated by phase lag index (PLI) in EEG signals in healthy older participants [healthy control (HC)] and patients with AD. We estimated signal complexity using multi-scale entropy. Utilizing a support vector machine, we compared the identification accuracy of AD based on functional connectivity at each frequency band and complexity component. Additionally, we evaluated the relationship between synchronization and complexity. The identification accuracy of functional connectivity of the alpha, beta, and gamma bands was significantly high (AUC 1.0), and the identification accuracy of complexity was sufficiently high (AUC 0.81). Moreover, the relationship between functional connectivity and complexity exhibited various temporal-scale-and-regional-specific dependency in both HC participants and patients with AD. In conclusion, the combination of functional connectivity and complexity might reflect complex pathological process of AD. Applying a combination of both machine learning methods to neurophysiological data may provide a novel understanding of the neural network processes in both healthy brains and pathological conditions.

## Introduction

With a growing aging population, we are awaiting an effective treatment strategy and an early diagnosis test for Alzheimer's disease (AD) (1–4). In AD, three main anatomical changes are observed: progressive neuronal death, neurofibrillary tangles, and senile plaques in widespread brain regions; moreover, recent progress of genome wide association studies have revealed the genes associated with AD (5, 6). For diagnosis, positron emission tomography (PET) imaging and magnetic resonance imaging (MRI) are widely used to detect neurotransmitter activity deficits and deposition of amy loid beta plaques, and brain atrophy, respectively (2–4). As other plausible diagnosis methods, tests based on temporal behaviors of neural activity, which are captured by electroencephalography (EEG), magnetoencephalography (MEG), and functional magnetic resonance imaging (fMRI), have been studied (7–13). Among these methods, those based on EEG are highly effective in clinical application, because they are cost-effective, widely available, and non-invasive (14, 15). The pathological progression of AD leads to cortical disconnection; consequently, in EEG signals, it alters functional connectivity measured by the degree of synchronization between different brain regions and complex behavior produced by the interactions among wide spread brain regions (9, 11, 12, 16–23).

To evaluate the complexity in the EEG signals in patients with AD, some studies have investigated an approach focusing on deterministic chaos and fractal dimension such as correlation dimension and Lyapunov exponent (24). These studies reported reduced complexity in the neural activity of patients with AD (8, 25–30). While, EEG dynamics plays a different role at each temporal-scale, such as memory function, cognitive function, and perceptual function in the theta, beta, and gamma bands, respectively (31). Therefore, an evaluation of the complexity of temporal-scale dependency in the EEG signals of patients with AD is an effective method. For this temporal-scale-specific complexity, several types of evaluation methods have been proposed, e.g., multiscale entropy based on sample entropy for course-grained time-series (32, 33), temporal-scale-specific fractal dimension expanded from Higuchi's fractal dimension (34), and maximum Lyapunov exponent and correlation dimension in band-specific EEG signals processed by wavelet transformation (35, 36). Particularly, our previous study using multi-scale entropy demonstrated that the complexity of EEG signals in patients with AD decreases at smaller (faster) temporal scales, but increases at larger (slower) temporal scales (37). In our study with the temporal-scale-specific fractal dimension, the reduced complexity is restricted to temporal scale regions faster and slower than the alpha band scale; moreover, the complexity around the alpha band scale exhibits high correlation with cognitive decline (34). Further, Adeli and colleagues revealed that alternation of complexity appears at the delta and theta bands in the eyes-open condition and at the delta, theta, and alpha bands by maximum Lyapunov exponent and correlation dimension analysis for band-specific EEG signals (35, 36).

Measuring coherence has long been used to evaluate functional connectivity in patients with AD, and it has revealed band-specific alterations of functional connectivity (38, 39). Wada et al. reported that resting-state functional connectivity reduces in the alpha and beta bands in AD (38). Sankari et al. showed that both enhancement and reduction are observed with frequency-band and spatial dependence (39). However, the studies with traditional synchronization indexes, typified as coherence measurements, correlation and mutual information, are influenced by volume conduction and can detect spurious synchronization (40, 41). To solve this problem, indexes for phase synchronization have been proposed, such as the synchronization likelihood (42), imaginary part of coherency (43) and phase lag index (PLI) (44). These indexes achieve fine temporal and spatial resolution for functional connectivity (42–44). By using this advantage and combining it with neuroimaging modalities that have high spatio-temporal resolution, such as magnetoencephalography (MEG), the alternations in functional connectivity for the whole brain network have been revealed (45–47). Stam et al. reported that functional connectivity in patients with AD estimated by PLI of MEG signals in the alpha and beta bands decreases; moreover, the clustering coefficient and path length of functional connectivity are reduced in the alpha band, i.e., the AD network approaches toward a random network (45). Engels et al. showed through PLI analysis of EEG signals that as AD progresses, the functional connectivity in the alpha band decreases and the hub structure shifts from the posterior to other regions in higher frequency bands (46).

Furthermore, recently, machine learning methods such as clustering algorithms and classification methods such as support vector machines (SVM), have been adopted to detect changes in functional connectivity in diseases (47–54). Yu et al. applied a method used for hierarchical clustering organization in minimum spanning trees on the functional connectivity of EEG signals in AD and frontotemporal dementia; they revealed disease-specific changes in brain network efficiency [Yu etal. (47)]. Khazaee et al. applied several graph measures such as degree, betweenness centrality, and local efficiency of the functional connectivity estimated by resting-state functional magnetic resonance imaging (fMRI) and showed that it accurately identified AD (54). Thus, machine learning approaches to study functional connectivity have been successfully applied to assess AD.

In contrast to these approaches, studies using a machine learning approach based on the complexity of brain activity have rarely been reported (55), despite EEG/MEG signals having features that can be used to identify AD. Within this context, it is important to compare the accuracy of identifying AD in machine learning approaches based on functional connectivity and those based on complexity. Therefore, in this study, we examined the functional connectivity estimated by PLI in EEG signals and the complexity estimated by MSE in healthy older people [healthy control (HC)] and patients with AD. We evaluated the identification accuracy of AD by using an SVM based on functional connectivity at each frequency-band and based on the complexity component. These identified characteristics were also evaluated.

## Materials and Methods

### Participants

The study consisted of 16 participants diagnosed with AD and 18 age- and sex-matched healthy control (HC) participants (34, 37). HC participants were functionally normal, independent in their daily lives, and did not take central-nervous-system-active medications. Patients with AD fulfilled the NINCDS-ADRDA work group criteria for probable AD (56), and the DSM-IV criteria for primary degenerative dementia and presenile onset. Moreover, to remove the other medical factors that induce dementia, the patients with AD were excluded based on neurological, serological, and neuroimaging [MRI and/or Computed Tomography (CT)] tests. The severity of AD in each patient was assessed by the functional assessment stages (FAST) (57) and a Japanese version of MMSE (58). The detailed information of the participants is presented in **Table 1**. Here, the sample size of the patients with AD is larger than that in our previous works (34, 37), because increasing the dataset as much as possible is required for SVM learning. All participants were medication-free, non-habitual drinkers, non-smokers, and right-handed. All participants provided informed consent before the initiation of the study. The study protocol was approved by the Ethics Committee of the Kanazawa University. All procedures of this study were performed in accordance with the Declaration of Helsinki.

**Table 1:** Physical characteristics in healthy older participants [healthy control (HC)] and Alzheimer's disease (AD) participants.

	HC participants	AD participants	*p* values
Male/female	7/11	5/11	0.72
Age (year)	59.3 (5.3, 55–66)	57.5 (4.7, 43–64)	0.31
MMSE score	NA	15.5 (4.7, 10–26)	NA
Assessment of AD	NA	NINCDS-ADRDA work group criteria for probable AD	NA
		DSM-IV criteria for primary degenerative dementia and presenile onset	
FAST assessment	NA	three (FAST3), seven (FAST4), and six (FAST5) patients.	NA

[Values represent mean (SD, range)]. FAST, functional assessment stages.

### EEG Recordings

The method for recording and pre-processing EEG data was established as reported in our previous study (37). During EEG recording, participants were seated in an electrically shielded, sound proofed recording room, and light was controlled. Standard scalp EEG electrodes were located in accordance with the International 10–20 System. In EEG recording, we used an 18-channel electroencephalogram (EEG-4518, Nihon-Koden, Tokyo, Japan) at 16 electrodes sites: Fp1, Fp2, F3, Fz, F4, F7, F8, C3, C4, P3, Pz, P4, T5, T6, O1, and O2, referenced to physically linked ear lobe electrodes. Eye movements were tracked using bipolar electro-oculography (EOG). The EEG signals were recorded with a 200 Hz sampling frequency, a time constant of 0.3, and a 1.5 to 60 Hz bandpass filter. The line noise at 60 Hz was removed by a notch filter. The impedance of electrode/skin conductance for each electrode was carefully controlled at less than 5 kΩ. EEG signals for each participant were measured for 10–15 min under the eyes closed resting condition. Using a video monitoring system, the vigilance state of the participant was visually inspected to ensure only epochs at eyes-closed wakefulness state (and not light sleep) were measured. EEG time-series segments recorded in the eyes-closed wakefulness state were identified by visual inspection of the EEG and EOG recordings. We considered that the participant was fully awake when predominant alpha activity appeared over the posterior regions, corresponding to fast eye movements in the EOG channel (59).

The data were stored on a magnetic optical disk for off-line analysis. Other pre-processing steps (i.e., filtering, artifacts removal, or data reconstruction) were avoided, because they may destroy the intrinsic dynamics of the data; epochs without artifacts were selected after a rigorous visual inspection. To evaluate long temporal dynamics, we initially prepared a single artifact-free, 60-s (12,000 data points) continuous epoch during the eyes-closed resting condition. Additionally, against the above dataset, 1,000 data points at the beginning and end of this epoch were removed to avoid the transition effect of the 1.5 to 60 Hz bandpass filter. MSE analysis was conducted against the continuous 50-s (10,000 data points) epoch. For PLI analysis, a long epoch length prevents identification of disease-specific changes, because the value becomes small with increasing epoch length (60) and vice versa. Furthermore, using a short epoch length cannot capture behaviors with slow frequency components. To balance them, for PLI analysis the continuous 50 s (10,000 data points) was divided into 10 epochs of 5 s (61, 62).

### Phase Lag Index

To measure phase synchronization, the characteristics of synchronous signals can be quantitatively estimated at different detection points by calculating the PLI. Firstly, the EEG signals were divided into 5 frequency bands: delta (2–4 Hz), theta (4–8 Hz), alpha (8–13 Hz), beta (13–30 Hz), and gamma (30–60 Hz). Each band-divided signal at time *t* and point *α* is represented by the phase *ϕa* (*t*) and the amplitude *Aa* (*t*) *via* the Hilbert transform. Subsequently, the difference in the phases ∆*ϕ_a__b_* (*t_i_*) observed between signals with two different detecting points *α* and *b* at time *t_i_* was written as Stam et al. (44)

(1) $$\text{Δ}\phi_{ab}\;\left(t_{i}\right)=\phi_{a}\;\left(t_{i}\right)-\phi_{b}\;\left(t_{i}\right)\;,$$

and

(2) $$\text{Δ}\phi_{\mathrm{mod}}\;\left(t_{i}\right)=\text{Δ}\phi_{ab}\left(t_{i}\right)\text{ mod }2\pi \;.$$

From Eq.(2), we obtained |∆ϕ_mod_(*t_i_*)|≤π. The PLI of signals between two observed points *α*–*b* for a number of signals *T* is defined as

(3) $$PLI_{ab}=\left|\frac{1}{T}\sum_{i=0}^{T}\text{sign}\left(\Delta \phi_{\text{mod}}\;(t_{i})\right)\right|\;,$$

where the PLI in Eq.(3) is moderately synchronous near 1.0, but is random near 0. From Eq.(1) and Eq.(2), the value of PLI in a case when signals with a common source are observed at different points becomes 0 because ∆*ϕ_ab_* (*t_i_*) is 0, and ∆*ϕ_mod_* (*t_i_*) = 0. In addition, the observation at a point located on the opposite side of the electric dipole has ∆*ϕ_ab_* (*t_i_*) = π in Eq.(1), where a signal source is assumed to follow the dipole model. Because this results in PLI*_ab_* = 0, the PLI also omits this signal.

We considered the averaged PLI of any electrode *α* through other electrodes *b* = 1, 2, ⋯, *K*(*b* ≠ *α*), which is called the node degree (ND), from Eq.(3) as

(4) $$NDa=\frac{1}{K-1}\sum_{b=1,b\neq a}^{K}\;\;{\text{PLI}}_{ab}\;,$$

where, *K* in Eq.(4) represents the total number of electrode, and has K = 16.

### Multi-Scale Entropy

In MSE analysis, the sample entropy (SampEn) is calculated by temporal-course grained time-series to evaluate the temporal scale dependency of complexity (32, 33). We define a coarse-grained time series from observed signals *x_i_* (*i* = 1, 2,⋯,*T*) over non-overlapping time segments as follows:

(5) $$y_{j,(\tau )}=\frac{1}{\tau }\sum_{i=(j-1)\tau +1}^{j\tau }x_{i}\;,\;\;\;\;1\leq j\leq \frac{T}{\tau }\;,$$

where is *τ* a scale factor. Subsequently, the MSE can be evaluated by calculating SampEn in terms of scale factor, which is described as

(6) $$\text{SampEn }\left(r,m\right)=-\ln\left[\frac{C_{m+1}\left(r\right)}{C_{m}\left(r\right)}\right]\;,$$

where *r* and *m* are the tolerance level and the length of the sequences, respectively. *C_m_* (*r*) in Eq.(6) is given as

(7) $$C_{m}=\sum_{i,j\in r,i\neq j}\frac{\left||Y_{i}^{m}-Y_{j}^{m}|\right|}{\left(T-m+1)(T-m\right)}\;,$$

where $\left||Y_{i}^{m}-Y_{j}^{m}|\right|$ indicates that it is counted when the distance between any two vectors $Y_{i}^{m}$ and $Y_{j}^{m}$ as the norm is less than *r*, $||Y_{i}^{m}-Y_{j}^{m}||=|y(i+l-1)-y(j+l-1){|}_{l=1,\cdots ,m}\leq r$ From Eq.(7), SampEn becomes 0 when the patterns remain the same and have no complexity, while SampEn becomes large when the patterns have high complexity.

### Statistical Analysis

For electrode-pair-wise group comparison of PLI between HC and AD groups, an independent two-tailed *t*-test was used. Here, *t*-statistical analysis was controlled by multiple comparison. Particularly, *t*-values corrected to *q* < 0.05, 0.01 were applied to PLI (600 *p* values: 120 electrode pairs × 5 bands) according to Benjamini–Hochberg false discovery rate (FDR) correction.

For the ND of PLI, repeated measures analysis of variance (ANOVA), with group (HC vs. AD) as the between-subject factor and electrode (16 electrodes from Fp1 to O2) as the within-subject factor, was performed to test for group differences at each band (delta to gamma bands). The Greenhouse-Geisser adjustment was applied to the degrees of freedom, and *α* two-tailed level of 0.05 was considered as statistically significant criteria in order to avoid type I error. To assess the significant main effect of group and the electrode-wise interactions, post-hoc *t*-tests were utilized. To control the multiple comparison, FDR correction was applied to the *t*-scores of ND (*q* < 0.05, 0.01) (80 *p* values: 16 electrodes × 5 bands).

For SampEn, repeated measures ANOVA with group (HC vs. AD) as the between-subject factor and electrode (16 electrodes form Fp1 to O2) and temporal scale (20 temporal scales) as within-subject factors, was performed to test for group differences. The Greenhouse-Geisser adjustment and a two-tailed *α* level of 0.05 were used as well as ND of PLI case. To assess the significant main effect of group and the interactions for electrode-wise and temporal-scale-wise, post-hoc *t*-tests were utilized. For multiple comparison, FDR correction was applied for the *t*-scores of SampEn (*q* < 0.05, 0.01) (320 *p* values: 16 electrode × 20 scales).

For identification of AD, a linear SVM based on the ND of PLI and SampEn was used. As pre-process for classification, by the principal component analysis, the principal components that are required to explain at least 90% variability of all components were chosen in order of the first principal component. SVM learning was conducted using these principal components. To examine the ability to classify HC and AD groups by SVM, we used receiver operating characteristic (ROC) curves that quantify the balance between sensitivity and specificity (63). Performance was evaluated by measuring the area under the ROC curve (AUC), which is an index for the overall identification accuracy. An AUC of 1.0 corresponds to perfect discriminating ability, while an AUC of 0.5 leads to random prediction. Here, 5-fold cross-validation was used.

To evaluate the relationship between synchronization and complexity, we used Pearson's correlation coefficient *R* between SampEn and ND of PLI. To control the multiple comparison, FDR correction was applied these *R*-scores (*q* < 0.05, 0.01) (1,600 *p* values: 16 electrodes × 5 bands × 20 scales).

## Results

### Phase Lag Index Analysis

**Figure 1:**
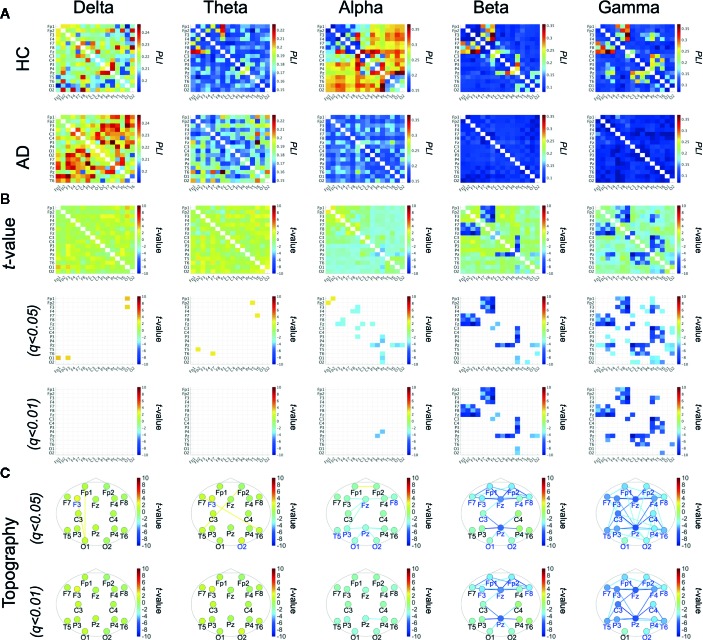
**(A)** Mean values of phase lag index (PLI) in the healthy control (HC) group and the Alzheimer's disease (AD) group. **(B)** *t*-scores for differences between the HC and AD groups (top parts) and *t*-scores passing through the criteria adjusted for false discovery rate (FDR) *q* < 0.05, *q* < 0.01 (corresponding to) *p* < 5.90 × 10^–3^), *p* < 6.73 × 10^–4^, respectively) (middle and bottom parts). **(C)** *t*-scores of PLI passing through the criteria adjusted for FDR: *q* < 0.05, 0.01 across the topography. *t*-scores for node degree (ND), where colored electrode labels correspond ones for passing through the criteria after adjustment FDR *q* < 0.05, *q* < 0.01 (corresponding to *p* < 0.0232 *p* < 2.51 × 10^–3^, respectively). Bluer (redder) colors represent the reduction (enhancement) of ND/PLI values in AD group.

**Figures 1A, B** show the mean PLI values for the HC and AD groups and their difference at each frequency band. *t*-tests with FDR correction in the bottom two rows of **Figure 1B** show significantly decreased PLI values in the AD group at the alpha, beta, and gamma bands. **Table 2** summarizes the repeated measures ANOVA test results for group differences in the ND of PLI. The significant group × electrode interactions and the main effect of group at alpha, beta, and gamma bands. **Figure 1C** shows a post-hoc *t*-test for the ND of PLI controlled by FDR correction and represents edges (electrode pairs) with significant group differences corresponding to **Figure 1B**. In post-hoc *t*-tests for ND, the ND of F8, Fz, P3, Pz, T5, O1, and O2 at the alpha band; the ND of electrodes except for C4, T5, P3, P4, and T6 at the beta band; and the ND of all electrodes at the gamma band passed through the criteria of *q* < 0.05 (corresponding to *p* < 0.0232). The ND of Fp1, Fp2, F3, F8, Fz, and Pz at the beta band and the ND of all electrodes except for O1 at the gamma band passed through the criteria of *q* < 0.01 (corresponding to *p* < 2.51 × 10^–3^). A significant reduction of pair-wised PLI in the AD group was mainly observed at the alpha, beta, and gamma bands among widespread regions.

**Table 2:** Repeated measures ANOVA results for the ND of PLI comparing HC and AD groups for each band.

Frequency band	Group effect	Group × node
delta	*F* = 2.18, *p* = 0.14	*F* = 0.83, *p* = 56
theta	*F* = 2.77, *p* = 0.10	*F* = 1.57, *p* = 0.13
alpha	***F* = 5.80, *p* = 0.02**	***F* = 3.86, *p* = 2.5 × 10^–3^**
beta	***F* = 12.49, *p* = 1.2 × 10^–3^**	***F* = 10.01, *p* = 0.00**
gamma	***F* = 27.78, *p* = 9.0 × 10^–6^**	***F* = 8.47, *p* = 0.00**

For clarity, comparisons with p < 0.05 are shown in bold.

### Multi-Scale Entropy Analysis

**Figure 2:**
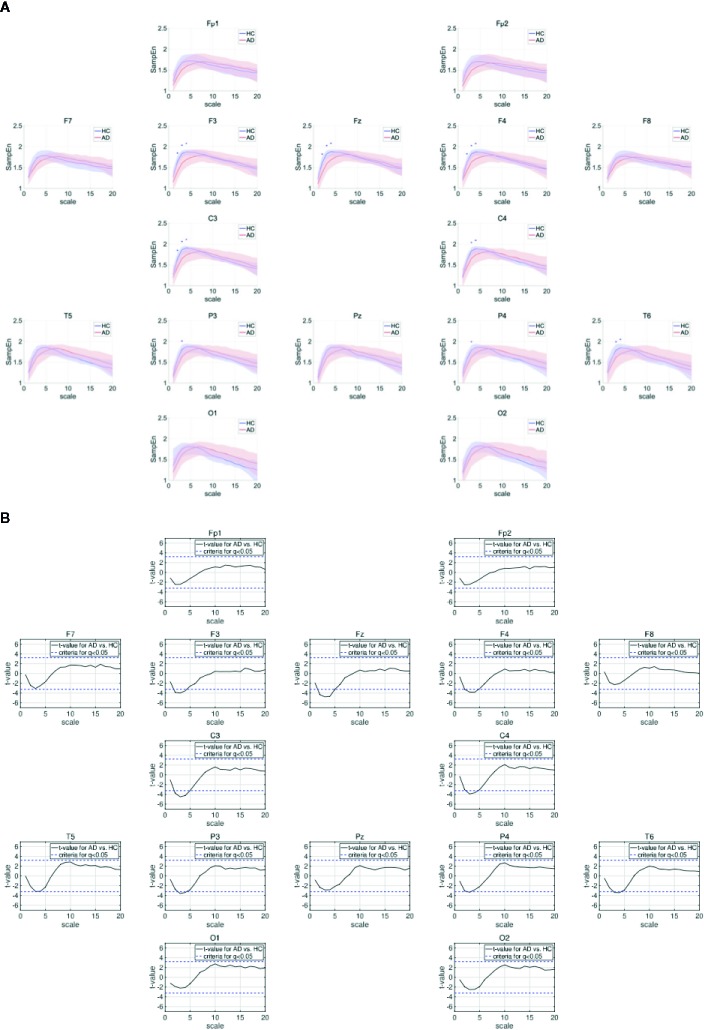
**(A)** Dependence of sample entropy (SampEn) on temporal scale. The blue + indicates the significant group difference satisfying the criteria after adjustment for FDR: *q* < 0.05 (corresponding to to *p* < 0.0029). Here, no significant group differences satisfying *q* < 0.01 were identified. **(B)** Dependence of *t*-values between SampEns for AD and ones for AD on temporal scale. Positive (negative) values indicate larger (smaller) SampEns for AD in comparison with HC. The *t*-values for criteria after adjustment FDR: *q* < 0.05 are represented by blue dashed lines (|*t*| > 3.23).

**Table 3** represents the results of repeated measures ANOVA results for the HC and AD groups. Significant group × scale interactions without the main effect of the group were observed. **Figure 2** shows the mean values of SampEn in the HC and AD groups and the results of the post-hoc *t*-tests. The results indicate a significant reduction of SampEn in AD (*q* < 0.05 corresponding to *p* < 0.0029) in the 1-5 scale ranges at F3 [scale 2: *t* = –3.85 (*p* < 5.2 × 10^–4^), scale 3: *t* = –3.99 (*p =* 8.35 *×* 10^–4^), scale 4*: t = –*3.54 *p* = 0.0025], F4 [scale 2: *t* = –3.82 *p* = 0.0029, scale 3: *t* = –3.79 (*p* = 0.0011), scale 4: *t* = –3.82 (*p* = 0.0011)], Fz [scale 2: *t* = –4.33 (*p* = 1.36 × 10^–4^), scale 3: *t* = –4.77 (*p* = 9.89 × 10^–5^), scale 4: *t* = –4.67 (*p* = 1.91 × 10^–4^)], C3 [scale 2: *t* = –3.75 (*p* = 6.94 × 10^–4^), scale 3: *t* = –4.48 (*p* = 2.13 × 10^–4^), scale 4: *t* = –4.24 (*p* = 5.07 × 10^–4^)], C4 [scale 3: *t* = –3.90 (*p* = 7.53 × 10^–4^), scale 4: *t* = –3.66 (*p* = 0.0015)], P3 [scale 3: *t* = –3.59 (*p* = 0.0011)], P4 [scale 4: *t* = –3.33 (*p* = 0.0022)], and T6 [scale 3: *t* = –3.36 (*p* = 0.0020), scale 4: *t* = –3.41 (*p* = 0.0018)].

**Table 3:** Repeated measures ANOVA results for Sample Entropy (SampEn) comparing HC and AD groups.

Group effect	Group × electrode	Group × scale	Group × electrode × scale
*F* = 0.17, *p* = 0.68	*F* = 1.12, *p* = 0.34	***F* = 6.67, *p* = 2.05 × 10^–3^**	*F* = 1.43, *p* = 0.163

For clarity, comparisons with *p* < 0.05 are shown in bold.

### Classification by the ND of PLI and MSE

AD was identified by linear SVM using significant reduction of the ND of PLI at the alpha, beta, and gamma bands. **Table 4** summarizes the accuracy of the classification between HC and AD by linear SVM based on the ND of PLI. Here, the ND values of all nodes were used at each band. The high ability to identify AD was confirmed (AUC = 10).

**Table 4:** Accuracy of classification between HC and AD by ND.

	Accuracy (%)	AUC	Size of principal components
Alpha band	100	1.0	4
Beta band	100	1.0	7
Gamma band	100	1.0	6

Here, the linear support vector machine (SVM) was used as the classification method and 5-fold cross-validation. Size of principal components means the size of components required to explain at least 90% variability of all components. AUC, area under the ROC curve.

In MSE analysis, in the scale ranges 1–5, a significant reduction of SampEn in the AD group was confirmed (see **Figure 2**). Against the mean value of SampEn in scales 1.5 at all electrodes, the linear SVM was adopted. **Table 5** shows the relatively high accuracy of identification (AUC = 81).

**Table 5:** Accuracy of classification between HC and AD by SampEn. AUC, area under the ROC curve.

	Accuracy (%)	AUC	Size of principal components
Mean SampEn in scale 1–5	73.5	0.81	3

### Correlation Between Synchronization and Complexity

**Figure 3:**
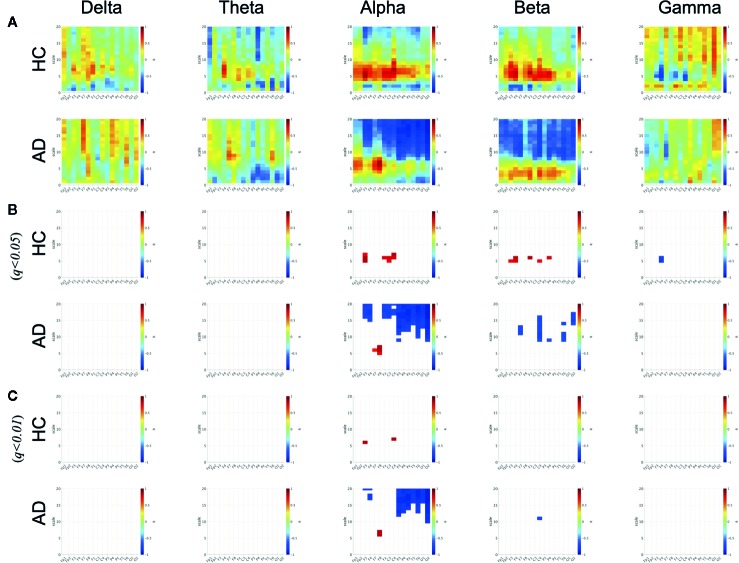
Correlation coefficient *R* between SampEn and the ND of PLI in HC and AD cases. There are significantly high positive and negative correlations passing through criteria of FDR (*q* < 0.05, 0.01) in alpha, beta, and gamma bands in HC and AD cases. **(A)** Correlation coefficient R. **(B)** R satisfying q<0.05. **(C)** R satisfying q<0.01.

**Figure 4:**
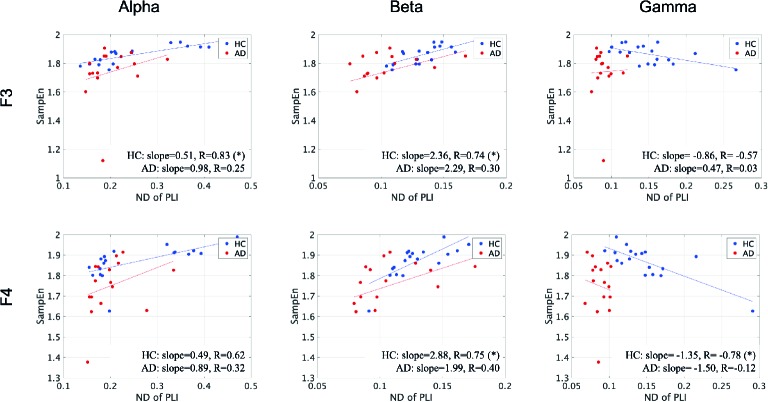
Scatter plots between SampEn at scale 5 and ND of PLI at alpha, beta, and gamma bands at F3 and F4 electrode in HC and AD cases. Here, the solid lines indicated the linear regression lines (blue: linear regression line for HC, red: linear regression line for AD). The correlation coefficient *R* [*R* value satisfying *q* < 0.05 is represented by (*)] and slope in the linear regression were described by text in figures. The slopes of correlation are different in HC and AD groups.

To evaluate the relationship between the ND of PLI and SampEn, the correlation coefficients between SampEn and the ND of PLI in the HC and AD groups were evaluated in **Figure 3**. The results show significantly high correlation passing through the criteria of FDR (*q* < 0.05, 0.01) in the alpha, beta, gamma bands. Particularly, a high positive correlation was observed at the frontal, central, parietal regions in the scale-range ≈ 5 in alpha and beta bands in the HC case. In the AD case, this correlation was observed at F7 and F8 in the alpha band. Moreover, a significant high negative correlation was observed at F4 at scales 5 and 6 in the gamma band in the HC case. In the AD case, this negative correlation was observed in a widespread region in the scale-range ≳ 10 in alpha and beta bands. **Figure 4** shows the scatter plots between SampEn at scale 5 and the ND of PLI at alpha, beta, and gamma bands at the F3 and F4 electrode in HC and AD cases. The slopes of correlation are different in HC and AD groups (The values of slope and *R* are represented in **Figure 4**).

## Discussion and Conclusion

In this study, we evaluated functional connectivity using PLI and complexity by measuring MSE in HC and AD groups. Significant reductions of PLI in the alpha, beta, and gamma bands and of SampEn at small (fast) temporal-scales were confirmed in AD group. Next, we classified the HC and AD groups by the linear SVM using the ND of PLI and SampEn averaged in the small temporal-scale range. We confirmed a significantly higher identification accuracy of the functional connectivity of the alpha, beta, and gamma bands (AUC: 10), and a sufficiently high identification accuracy of complexity (AUC: 81). Furthermore, we evaluated the relationship between functional connectivity and complexity, and found various temporal-scale-and-regional-specific dependencies in both HC participants and patients with AD.

Regarding functional connectivity in the EEG/MEG signals in patients with AD, many previous studies have reported a reduction in functional connectivity at the alpha, beta, and gamma bands (38, 45, 46, 64). Further, recent studies of AD pathology have revealed that the reduction in functional connectivity is caused by neuroinflammation and deposition of amyloid-*β* and tau proteins (65–67). Similar reductions in functional connectivity were also observed in this study. Regarding the complexity of the EEG/MEG signals in patients with AD, many studies have reported alternations of temporal behaviors (23); particularly, the reduction in the complexity of the EEG/MEG signals in patients with AD (8, 22, 25–30). Analysis of the indexes for temporal-scale-dependent complexity has shown that this reduction of complexity especially concentrates in fast wave components (34, 36). Studies of neurotransmitter changes in AD have reported that dysfunction of the gamma-aminobutyric acid (GABA) signaling system, which is caused by the deposition of amyloid- *β* and tau proteins, leads to reduced oscillatory gamma band activity (68–70). The impairment of gamma oscillatory activity might lead the complexity at faster temporal scales more than slower temporal scales (34, 36). The results obtained with MSE analysis are congruent with these findings.

We must consider the reason the changes in functional connectivity exhibited significant regional specificity, while the complexity did not. As a plausible explanation, it is assumed that synchronization is determined by the interaction between brain regional pairs, while the characteristics of complexity are produced by interactions among wide-spread brain regions (9). Therefore, it might be difficult when complexity exhibits regional specificity. Model-based studies regarding the relationship between complex neural behavior and topological features of the whole network support these findings (71–75). In the classification of HC and AD by SVM, the ND or the mean SampEn averaged in fast temporal-scale region at 16 electrodes were used. Higher disease-specific regional dependency might enhance the accuracy of identification of AD, because classification can be conducted in a feature space with larger dimensions. Therefore, it can be assumed that SVM based on the ND of PLI with higher regional dependency exhibited higher identification accuracy in comparison with one based on SampEn.

Furthermore, we must discuss the necessity of focusing on the complexity of EEG/MEG signals to identify AD. Cortical disconnection, which is induced by the pathological progression of AD, leads to impairment in the interaction between different brain regions; consequently reducing functional connectivity and complexity (9, 11, 12, 16, 17, 19–21). The positive correlation between them (see **Figure 3**) can be supported by these findings. Moreover, the slopes in these correlations were different between the HC and AD groups (see **Figure 4**). Therefore, this relationship between complexity and functional connectivity could be used for diagnosis of AD. Not only positive correlation but also negative correlation was confirmed in **Figure 3**. According to nonlinear dynamical theory, it is known that non-linear coupled oscillations exhibit enhancement of complexity by emergence of a chaotic state during the process of reducing the efficiently strong coupled strength for the state of complete synchronization [reviewed in (76). This induced enhancement is attributed to perturbations to the stable orbit from each oscillation's behavior (77, 78). Synchronization decreases during the process of decreasing coupled strength, and complexity enhances. The negative correlation observed in this study could be interpreted by this mechanism. These findings suggest that a combination of functional connectivity and complexity might reflect the complex pathological process of AD.

To investigate if the high heterogeneity of age and severity in the patients with AD affected classification, we performed repeated measures ANOVA in the AD group. For MSE analysis, the repeated measures ANOVA was performed with age [high age vs. low age (these groups were divided by median of AD age distribution = 59.5 years)] and severity (FAST scale score: 3, 4, and 5) as between-subject factors, and electrode and scale factor as within-subject factors. The results showed no significant high main effect and interactions. For the ND of PLI at each frequency band, the repeated measures ANOVA was performed with age (high age vs. low age) and severity (FAST scale score: 3, 4, and 5) as between-subject factors, and electrode as a within-subjects factor. The results showed that severity did not demonstrate any significant main effects or interaction. In contrast to the MSE case, age showed a significant high main effect in the theta [*F* = 5.86 (*p* = 0.0029)], beta [*F* = 6.16 (*p* = 0.026)], and gamma [*F* = 8.9 (*p* = 9.84 × 10^–3^)] bands, and a significant high interaction between age vs. electrodes at the beta [*F* = 4.73 (*p* = 1.59 × 10^–3^)] and gamma [*F* = 5.10 (*p* = 2.41 × 10^–4^)] bands. Further, in larger AD groups, the severity-dependent effect may appear in both PLI and MSE cases. Therefore, to consider these effects in classification by SVM, a larger sample size is necessary.

This study has limitations that must be considered. The data set of the HC and AD groups used in this study can completely identify AD using SVM and the ND of PLI. Therefore, the effect of enhancing identification accuracy by combining with components of SampEn cannot be evaluated. To evaluate this effect, the classification of the severity of AD (FAST 3, 4, and 5) may be appropriate. However, for this evaluation, the size of data set used in this study was too small for SVM learning. Moreover, the AD group had high heterogeneity of age and severity, which could have influenced the accuracy of the classification by SVM. However, the sample size of the AD group was too small to quantify this influence. In future studies, we will evaluate these issues using a larger data set of AD EEG signals. Another limitation of this study is that the EEG signals do not necessarily reflect neural activity directly under the electrodes; the spacial resolution of the 16 electrodes used in this study was too low to identify the complex functional connectivity structures relating to AD pathology. Therefore, the use of neuroimaging modalities with more precise and higher spatial resolution, such as MEG and cortical source localization, may provide the necessary spatial information. Finally, data sets of other pathological conditions, such as schizophrenia and autism spectrum disorder, and healthy aging must be evaluated by the machine learning method used in this study.

In conclusion, we confirmed that the identification accuracy of SVM based on functional connectivity was significantly high, and the identification accuracy of SVM based on complexity was sufficiently high. Moreover, the combination of functional connectivity and complexity might reflect the complex pathological process of AD. Although some limitations must be considered, applying a combination of machine learning methods to neurophysiological data may provide a novel understanding of the neural network processes in both healthy brains and pathological conditions.

## Data Availability Statement

The datasets generated for this study will not be made publicly available because the informed consent did not include the declaration regarding publicity of clinical data. Requests to access the datasets should be directed to the corresponding author.

## Ethics Statement

The studies involving human participants were reviewed and approved by the Ethics Committee of the Kanazawa University. The patients/participants provided their written informed consent to participate in this study.

## Author Contributions

SN, TY, TT, MK, and HN conceived the methods. SN, TY, SK, and TT analyzed the results, wrote the main manuscript text, and prepared all the figures. MK conducted the experiments. All authors reviewed the manuscript.

## Funding

This work was supported by JSPS KAKENHI for Early-Career Scientists (grant number 18K18124) (SN) and for Scientific Research (C) (grant number 18K11450) (TY).

## Conflict of Interest

The authors declare that the research was conducted in the absence of any commercial or financial relationships that could be construed as a potential conflict of interest.
